# Effects of Infusion and Decoction Mashing on Carbohydrate Structure, Fermentation Behavior, and Volatile Compound Profiles in Wheat Beer

**DOI:** 10.3390/foods15173163

**Published:** 2026-09-07

**Authors:** Yuquan Wu, Juan Du, Meiqi Li, Yanting Yin, Guixin Wang, Song Wang, Hongchen Fan, Guoqi Shang, Xiaohui Hu

**Affiliations:** 1 College of Food Engineering, Harbin University of Commerce, Harbin 150028, China; 2 Budweiser Harbin Brewery Co., Ltd., Harbin 150060, China; 3 Jiangsu Key Laboratory for Recognition and Remediation of Emerging Pollutants in Taihu Basin, School of Environmental Science and Engineering, Wuxi University, Wuxi 214105, China; huxiaohui@cwxu.edu.cn

**Keywords:** carbohydrate structure, fermentable sugars, mashing strategy, multivariate analysis, volatile aroma compounds, wheat beer

## Abstract

Beer flavor depends on the composition and structural characteristics of fermentable carbohydrates generated during mashing. Although mashing strategy is known to influence wort composition, the mechanistic relationship between mashing-induced variations in carbohydrate structure and subsequent yeast metabolism and flavor formation remains poorly understood. This study aimed to compare the effects of two mashing procedures, infusion mashing (IMM) and decoction mashing (DMM), on carbohydrate structure, fermentation behavior, and flavor attributes of wheat beer. The physicochemical properties were analyzed using standard methods; organic acids, amino acids, and volatile compounds were analyzed using chromatographic techniques combined with multivariate analysis. Instrumental aroma and taste characteristics were further assessed using electronic nose and electronic tongue systems. Compared with IMM, DMM wort contained numerically higher proportions of low-degree-of-polymerization carbohydrates and higher amino acid concentrations. These compositional differences were associated with divergent fermentation kinetics and distinct volatile and nonvolatile metabolite profiles in the finished beers. DMM was associated with higher concentrations of medium-chain fatty acid ethyl esters, whereas IMM was associated with higher concentrations of acetate esters, higher alcohols, and aldehydes, consistent with faster fermentation and enhanced amino acid catabolism. Multivariate analysis confirmed differentiation in volatile profiles and instrumental aroma characteristics between the two beers. Together, these results suggest an association of mashing strategy with variations in carbohydrate structure, fermentation behavior, and volatile compound profiles in wheat beer. These findings offer a framework for the process-level control of beer flavor.

## 1. Introduction

Wheat beer has gained increasing attention in recent years, driven by the rapid expansion of the craft beer market [1]. Typically brewed with a substantial proportion of wheat malt or unmalted wheat, wheat beer is distinguished by its characteristic turbidity, creamy mouthfeel, and fruity-spicy aroma profile [2,3]. These sensory attributes depend heavily on the fermentable sugars, amino acids, and aroma precursors generated during mashing [2]. Wheat beer differs from barley-based beer in several compositionally significant aspects. Wheat malt typically contains higher concentrations of protein, β-glucans, and arabinoxylans, which influence wort viscosity, yeast nutrition, foam stability, and mouthfeel [4]. These compositional features may interact with mashing conditions to modulate the final beer flavor profile, yet the specific contribution of mashing strategy to carbohydrate structure and flavor in wheat beer remains underexplored.

Mashing activates a suite of endogenous enzymes—*α*-amylase, *β*-amylase, limit dextrinase, proteases, and *β*-glucanases—whose coordinated action determines the structure and distribution of fermentable sugars, dextrins, and free amino nitrogen in wort [5]. These constituents, in turn, govern yeast metabolism, fermentation kinetics, and the formation of flavor-active compounds. Multiple mashing parameters have been found to influence beer quality: temperature profiles and rest durations affect ethanol yield, attenuation, and foam stability [6,7,8]; mash pH alters the biochemical composition and staling properties of sweet wort; and debranching enzyme activity shifts the balance between fermentable sugars and dextrins, with direct consequences for fermentation [9]. Despite these advances, most studies have centered on general physicochemical parameters or process efficiency, while the compositional and flavor-related outcomes of various mashing strategies remain to be elucidated.

The two dominant mashing strategies—infusion mashing and decoction mashing—differ markedly in thermal profile and process logic. Infusion mashing is valued for its operational simplicity, shorter processing time, and lower energy demand [10]. Decoction mashing, in which a portion of the mash is boiled and returned to the main vessel, enhances starch gelatinization, promotes enzymatic hydrolysis, and can improve wort stability and flavor complexity, albeit at the cost of greater energy consumption and process complexity [11,12]. Both methods are widely practiced across brewing scales [13]. However, how these two mashing strategies differentially shape the carbohydrate structure of wort and thereby modulate yeast metabolism and flavor formation has received comparatively less attention. A more detailed understanding of these relationships would support the rational, process-level control of beer flavor quality.

Prior comparative studies on infusion and decoction mashing have largely addressed process efficiency, antioxidant capacity, or broad sensory quality [11,14]. A few have examined how the two strategies alter the structural characteristics of fermentable sugars and how such structural variations propagate through yeast metabolism to influence volatile flavor formation in wheat beer.

Beer flavor arises from a complex ensemble of volatile and nonvolatile compounds, including organic acids, free amino acids, higher alcohols, esters, aldehydes, and phenolics. Headspace solid-phase microextraction coupled with gas chromatography–mass spectrometry (HS-SPME-GC-MS) enables detailed volatile profiling [15], while electronic nose and electronic tongue systems provide rapid, objective pattern recognition of aroma- and taste-related attributes [16]. An integrated application of these complementary techniques offers a means for tracing mashing-induced compositional changes and sensory-relevant outcomes.

The present study was conducted to investigate how infusion and decoction mashing influence fermentable sugar structure and flavor development in wheat beer. Even when the same yeast strain and fermentation conditions are used, beers obtained using different mashing protocols can differ appreciably in alcohol content, flavor profiles, and mouthfeel; these differences are likely rooted in the carbohydrate composition of wort, particularly the proportions of fermentable sugars and dextrins [17]. Fermentable sugar profiles, dextrin content, and amino acid availability collectively shape yeast metabolic activity and thus affect ethanol production, formation of aroma compounds, and sensory perception [18]. Mashing represents a cost-effective and scalable intervention point for flavor regulation; however, translating this opportunity into practice requires a clearer understanding of how mashing-driven variations in carbohydrate structure propagate through yeast metabolism. To address this gap, we combined physicochemical analyses, organic acid and free amino acid profiling, volatile compound characterization using HS-SPME-GC-MS, electronic nose and electronic tongue measurements, and multivariate statistics. Our results suggest associations between mashing strategy, carbohydrate structure, fermentation dynamics, and volatile compound profiles in wheat beer, and offer a basis for process-level flavor management in brewing. We hypothesized that IMM and DMM would produce worts with different carbohydrate chain-length distributions, and these differences in carbohydrate structure would be associated with distinct fermentation kinetics and volatile compound profiles in the finished beers.

## 2. Materials and Methods

### 2.1. Materials

The brewing yeast strain *Saccharomyces cerevisiae* WB-06 was purchased from Fermentis (Lesaffre Group, Marcq-en-Barœul, France). Barley malt and wheat malt were obtained from Malteurop (Baoding) Malting Co., Ltd. (Baoding, China). Herkules hops were supplied by Super Malt (Beijing) Trading Co., Ltd. (Beijing, China). Sodium hydroxide, methanol, phosphoric acid, and other chemicals were obtained from Tianjin Tianli Chemical Reagent Co., Ltd. (Tianjin, China). All reagents were of analytical grade unless otherwise specified. The key quality parameters of the malt used in this study were as follows: moisture, 4.45%; extract, 81% (dry basis); diastatic power, 120° WK; total protein, 12.13% (dry basis); free amino nitrogen (FAN), 108 mg/100 g (malt, dry basis); color, 4.80 EBC; *β*-glucan, 72 mg/L (congress wort); viscosity, 1.44 mPa·s (congress wort); pH, 5.92; and acidity, 1.17 mL of 0.1 mol/L NaOH per 100 mL (congress wort). All malts were from a single production batch.

### 2.2. Beer Brewing Procedure

#### 2.2.1. Malt Milling

Barley malt and wheat malt were mixed in a ratio of 6:4 (*w*/*w*) to obtain 11 kg of grist. Prior to milling, 8% (*w*/*w*) water was applied to the barley malt to condition the husk. The malt mixture was then coarsely milled using a malt mill (B-400; BUCHI Labortechnik AG, Flawil, Switzerland).

#### 2.2.2. Mashing

**Figure 1:**
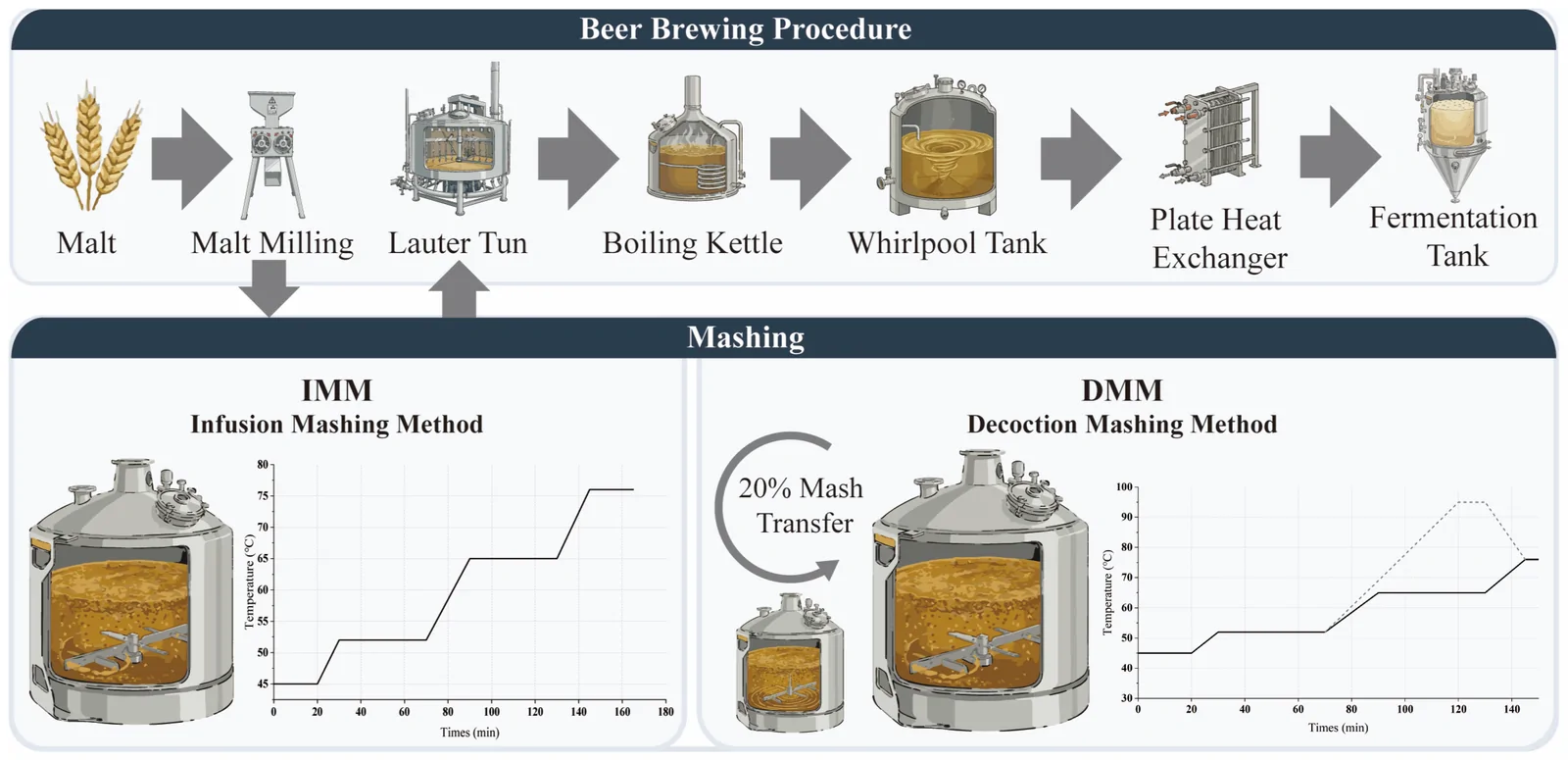
Schematic diagram of the brewing process and mashing strategies (IMM and DMM).

Two mashing strategies were compared: the infusion mashing method (IMM) and the decoction mashing method (DMM) (Figure 1). Mashing was conducted under atmospheric pressure without adding processing aids or exogenous enzymes.

For IMM, 50 L of water was added to the mash tun and heated to 45 °C before introducing the milled malt. The mash was held at 45 °C for 20 min, heated to 52 °C for 10 min and held for 40 min, then heated to 62 °C for 10 min and held for 10 min, followed by heating to 65 °C for 6 min and holding for 50 min. The temperature was then raised to 72 °C for 8 min and held for 10 min, and finally increased to 76 °C for 6 min to complete mashing. The mash was subsequently transferred to the lauter tun.

For DMM, the temperature program was identical to IMM except that approximately 20% of the mash was transferred to a separate decoction kettle at the 52 °C rest stage and subjected to an additional boiling step (95 °C, 10 min) before being returned to the main mash. No exogenous enzymes or processing aids were added during mashing, and all mashing procedures were conducted under ambient atmospheric conditions. Each mashing treatment was performed in three fully independent brewing trials. Identical raw materials (the same batch of barley malt and wheat malt, identical grist composition, and the same total water volume) were used for both IMM and DMM treatments. Therefore, any difference in the original extract between the two treatments reflected the inherent difference in extract recovery efficiency attributable to the mashing strategy itself, rather than to an uncontrolled experimental variable. No exogenous enzymes, processing aids, or extract adjustment procedures were applied during mashing or wort preparation. The pH of the mash was monitored but not actively adjusted during mashing; the natural mash pH stabilized at approximately 5.4 for both treatments. The actual heating rate was approximately 0.8 °C/min, and thermal uniformity was maintained by continuous low-speed agitation. The execution order of IMM and DMM trials was randomized across the three independent replicates to minimize systematic bias.

#### 2.2.3. Wort Filtration and Boiling

Before wort transfer, hot water at 78 °C was added to the lauter tun to preheat the filter plates. After transferring the mash, the wort was allowed to rest for 10 min to form a natural filter bed. The wort was then recirculated until clear and subsequently transferred to the boiling kettle. During transfer, the temperature was gradually increased to 90 °C. After filtration, the wort was heated to 98 °C and boiled. Herkules hops (5 g) were added at the start of boiling. The total boiling time was 60 min.

#### 2.2.4. Whirlpool Clarification and Fermentation

After boiling, the wort was transferred to a whirlpool tank and allowed to stand for 15 min to facilitate trub sedimentation. The clarified wort was then cooled to 18 °C using a plate heat exchanger (BR0.6; Shanghai Nanhua Transducer Manufacture Co., Ltd., Shanghai, China) and transferred to a fermentation tank. Oxygen was supplied prior to pitching to a dissolved oxygen concentration of 8 mg/L, and the wort was inoculated with WB-06 wheat beer dry yeast at a pitching rate of 1.2 × 10^7^ viable cells/mL (corresponding to approximately 1.2 g of active dry yeast per liter of wort, based on an estimated viable cell count of 2 × 10^10^ cells/g; approximately 30 g per 50 L batch). Yeast viability, determined by methylene blue staining prior to pitching, was >99.5%. The working volume of the fermenter was approximately 50 L, yielding approximately 40 L of wort per batch.

Fermentation was conducted at 21 °C for 7 days. After oxygenation to 8 mg/L dissolved oxygen, the fermenter was left unsealed (open to the atmosphere) in the first 24 h to permit aerobic respiration and yeast biomass accumulation. The fermenter was then sealed after 24 h to allow anaerobic fermentation to proceed. At the end of fermentation, the beer was cooled to 0 °C and stored for 48 h prior to further analysis. The three independent brewing trials referred to three fully separate brews conducted on different days using fresh raw materials each time, not technical triplicates from a single brew.

### 2.3. Methods

#### 2.3.1. Chain-Length Distribution and Molecular Weight Analysis

The mash samples were collected at the end of mashing, immediately cooled on ice to quench all enzymatic reactions, and stored at −20 °C until analysis. Fermented beer samples were collected after fermentation. All samples were centrifuged at 4000× *g* for 10 min using a centrifuge (CL5; Xiangyi Laboratory Instrument Development Co., Ltd., Harbin, China) to remove suspended solids. The supernatants were collected and freeze-dried using a lyophilizer (SCIENTZ-50F/A; Ningbo Scientz Freeze Drying Equipment Co., Ltd., Ningbo, China) at −50 °C under vacuum to obtain dry powder samples.

For chain-length distribution analysis, 10 mg of the freeze-dried powder was dissolved in 2 mL of ultrapure water and heated in a boiling water bath for 30 min to ensure complete dissolution. The solution was centrifuged at 10,000× *g* for 1 min, and the supernatant was collected, diluted appropriately, and filtered through a 0.22-μm aqueous membrane filter. Prior to HPAEC-PAD analysis, the dissolved samples were debranched using isoamylase (EC 3.2.1.68) to release the constituent linear chains from branched dextrins. The debranching reaction was performed at 100 U/mg of substrate in 50 mM sodium acetate buffer (pH 5.5) at 50 °C for 24 h, following the protocol described for debranching-based chain-length distribution analysis. The reaction was terminated by heating in a boiling water bath for 10 min, and the reaction mixture was centrifuged at 10,000× *g* for 5 min. The supernatant was collected, diluted appropriately, and filtered through a 0.22-μm aqueous membrane filter before injection [19,20]. The mobile phase comprised 250 mM NaOH and 1 M sodium acetate at a flow rate of 0.5 mL/min, and the column temperature was maintained at 35 °C. Individual maltooligosaccharide standards (DP1-DP13) were used to establish retention times and detector response factors. The concentration of each oligosaccharide fraction was calculated by applying the corresponding response factor to the measured peak area, thereby correcting for the degree of polymerization (DP)–dependent detector response of HPAEC-PAD. The relative proportion of each DP fraction was calculated as the corrected peak area of that fraction divided by the summed corrected peak areas of all detected fractions.

For molecular weight (Mw) analysis, 10 mg of freeze-dried powder was dissolved in 2 mL of dimethyl sulfoxide (DMSO). The mixture was sealed and heated in a boiling water bath with magnetic stirring overnight to ensure complete dissolution, and then filtered through a 0.22-μm organic membrane filter. Mw distribution was determined using a gel permeation chromatography (GPC) system equipped with two columns in series (WAT044205, Mw 5 × 10^3^–10^7^, and WAT054494, Mw 102–104) [21]. The mobile phase comprised 99.5% (*w*/*w*) DMSO and 0.5% (*w*/*w*) LiBr at a flow rate of 0.5 mL/min, and the column temperature was maintained at 50 °C. Pullulan standards with known Mw values were used for column calibration, and the weight-average Mw of each sample was calculated from the resulting calibration curve.

#### 2.3.2. Physicochemical Properties of Beer

**Figure 2:**
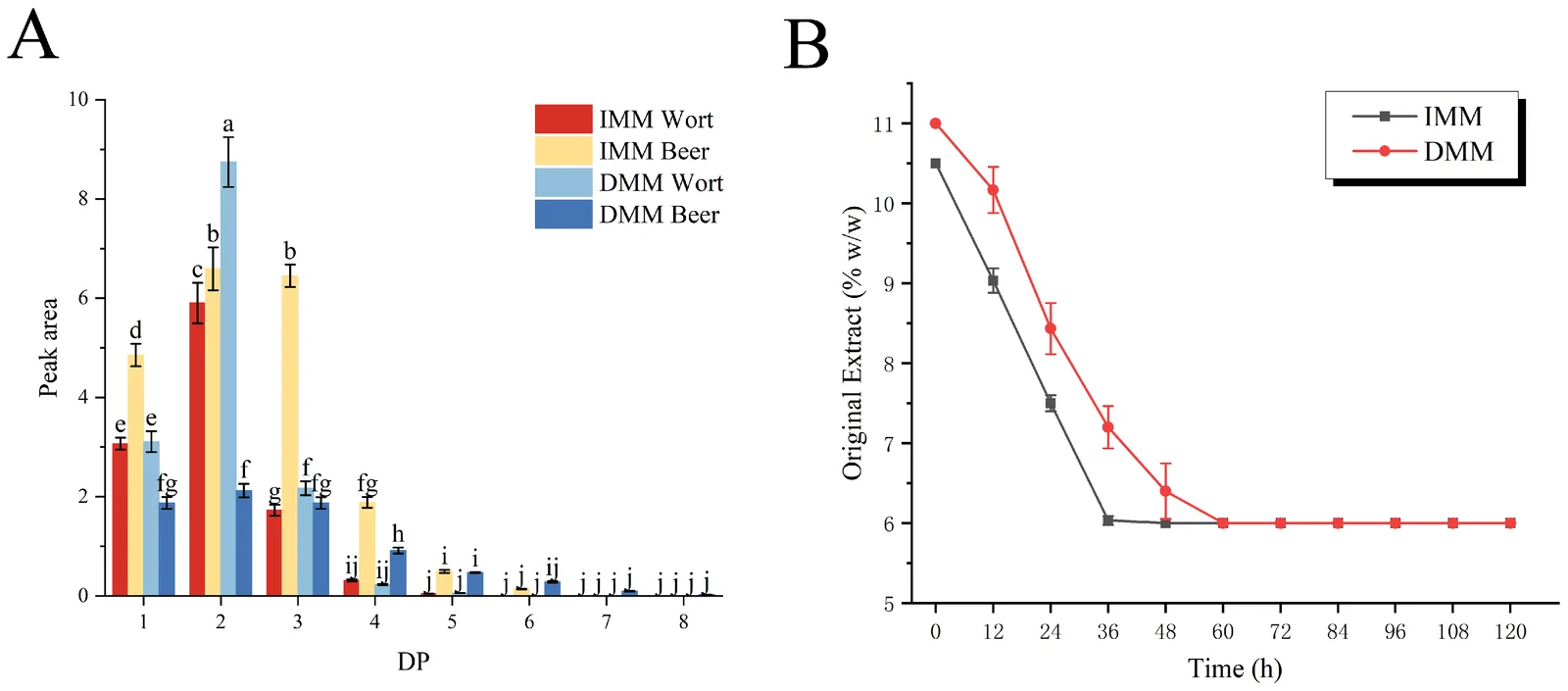
Effects of IMM and DMM on (**A**) DP distribution and (**B**) fermentation rate of yeast. There are significant differences in the representation of different letters (*p* < 0.05).

The soluble solids content of the fermenting wort was monitored every 12 h throughout fermentation using a handheld refractometer (HB-111ATC; Nanbei Instrument Co., Harbin, China). As the accumulation of ethanol during fermentation alters the refractive index, the refractometer readings (expressed as °Brix) do not directly represent true sugar or extract concentration after the onset of fermentation. The readings were therefore used solely to monitor the relative changes in soluble solids content over time (Figure 2B), and were not interpreted as absolute extract or sugar concentrations. Final beer physicochemical parameters (Table 1) were determined using an automatic beer analyzer (DMA 5000 M; Anton Paar (Shanghai) Trading Co., Ltd., Shanghai, China), which provides ethanol-corrected measurements. After fermentation, the beer samples were analyzed according to the Chinese national standard GB/T 4928-2026 [22] (Method for analysis of beer; Standardization Administration of China, 2026). The alcohol content, original extract, real extract, real degree of fermentation, pH, color, and caloric content were measured using the automatic beer analyzer (DMA 5000 M). Total acidity was determined by the pH-electrode potentiometric titration method (Method 2) of GB 12456-2021 [23] (National Food Safety Standard—Determination of Total Acids in Food): The sample was titrated with 0.1 mol/L sodium hydroxide standard solution to an endpoint pH of 8.2, and total acidity was expressed in mL/100 mL. The concentrations of free and total diacetyl and 2,3-pentanedione were determined by gas chromatography according to Appendix C.6 of GB/T 4928-2026 (Diacetyl, gas chromatographic method), using an electron capture detector with headspace sampling and 2,3-hexanedione as the internal standard. The concentrations of free vicinal diketones (VDKs) were measured without prior heating, whereas the concentrations of total VDKs (including precursor conversion) were measured after heating the sample at 60 °C for 90 min.

#### 2.3.3. Concentrations of Organic Acids and Free Amino Acids

The concentrations of organic acids were determined by high-performance liquid chromatography (HPLC) as previously described [24] with slight modifications. The analysis was performed on an Agilent 1260 HPLC system (Agilent Technologies, Santa Clara, CA, USA) equipped with an InfinityLab Poroshell 120 EC-C18 column (4.6 × 150 mm, 4 μm). The injection volume was 5 μL, the column temperature was maintained at 30 °C, and detection was carried out at 210 nm. The mobile phase comprised (A) 0.1% phosphoric acid and (B) methanol. The flow rate was 0.5 mL/min, and the gradient program was as follows: 2% B from 0 to 10 min, increased to 40% B from 10 to 16 min, and returned to 2% B from 16 to 19 min.

The concentrations of free amino acids were analyzed using a Sykam S433D amino acid analyzer (Sykam GmbH, Munich, Germany) equipped with a K06/Na ion-exchange column. The amino acids were quantified by post-column ninhydrin derivatization as previously described [25]. The mobile phases comprised sodium citrate buffers: A (0.12 N, pH 3.45) and B (0.20 N, pH 10.85). The flow rate was 0.45 mL/min for the elution pump and 0.25 mL/min for the derivatization pump. The system pressure was maintained at 30–40 bar, and detection was performed at 570 and 440 nm. The column temperature program was as follows: held at 58 °C until 22 min, increased to 74 °C between 22 and 26 min, held at 74 °C until 47 min, decreased to 58 °C between 47 and 53 min, and held at 58 °C until the end of the run. The total analysis time was 58 min.

#### 2.3.4. Concentrations of Volatile Compounds

The concentrations of volatile compounds in beer samples were analyzed using HS-SPME-GC-MS.

Prior to analysis, the beer samples were gently degassed by magnetic stirring for 15 min to minimize the loss of volatile compounds. An aliquot of 10 mL of the beer was transferred into a 20 mL headspace vial, and 3.2 g of sodium chloride was added to enhance the volatilization of aroma compounds. 4-Fluorobenzaldehyde (50 mg/L) was added as an internal standard. The vial was equilibrated in a water bath at 40 °C for 10 min. A divinylbenzene/carboxen/polydimethylsiloxane SPME fiber (50/30 μm, 1 cm) was exposed to the headspace for 60 min. Thermal desorption was performed in the GC injector at 270 °C for 60 s.

GC-MS analysis was carried out on an Agilent 7890 gas chromatograph (Agilent Technologies, Inc., Santa Clara, CA, USA) coupled to a LECO Pegasus BT mass spectrometer (Laboratory Equipment Corporation, St. Joseph, MI, USA). A DB-Wax capillary column (30 m × 0.25 mm × 0.25 μm) was used for chromatographic separation, with helium as the carrier gas at a constant flow rate of 1.0 mL/min. The injector was operated at 270 °C in splitless mode. The oven temperature program was as follows: held at 40 °C for 5 min, increased to 120 °C at the rate of 3.5 °C/min, and then raised to 215 °C at the rate of 5 °C/min and held for 10 min [26].

MS detection was performed in electron ionization mode at 70 eV. The ion source temperature was 230 °C, and mass spectra were recorded over *m*/*z* 35–300.

Volatile compounds were identified by matching their mass spectra against the National Institute of Standards and Technology (NIST) and Wiley mass spectral libraries. A compound was considered positively identified when both the forward (Similarity) and reverse (Reverse) mass spectral match factors were ≥800. The NIST library retention index (Lib. RI) recorded for each match was also used as a supplementary reference for identification, and the identification confidence level of each compound was assigned based on the match factors.

Semi-quantitative analysis was performed using 4-fluorobenzaldehyde as the internal standard. The concentration of each volatile compound was expressed as 4-fluorobenzaldehyde equivalents (μg/L), calculated as (peak area of compound/peak area of internal standard) × concentration of the internal standard.

A procedural blank (ultrapure water processed identically to beer samples) was analyzed with each batch to identify potential contaminants or carryover. The stability of the SPME fiber and GC-MS system was monitored by injecting a quality control sample at the beginning and end of each analytical sequence. Compounds were identified by mass spectral matching against the NIST and Wiley libraries. The NIST library retention index (Lib. RI) recorded for each match was also reported as a supplementary reference. The Similarity, Reverse, and Probability values, together with the Lib. RI, for each compound are listed in Table A1. Quantitative values were reported as semi-quantitative concentrations in 4-fluorobenzaldehyde equivalents (μg/L), calculated as (peak area of compound/peak area of internal standard) × concentration of the internal standard. As compound-specific response factors were not determined, these values should be interpreted as relative comparisons between treatments rather than as absolute concentrations.

#### 2.3.5. Electronic Nose Analysis

The aroma profiles of wheat beer samples were evaluated using a commercial electronic nose system equipped with an array of 18 metal oxide semiconductor sensors and an intelligent pattern recognition system (Shanghai Baosheng Industrial Development Co., Ltd., Shanghai, China). For each measurement, 10 mL of beer was transferred into a sealed headspace vial and equilibrated at 40 °C for 60 min. Each beer sample was measured in triplicate, and all samples were analyzed in a single experimental session to minimize instrumental drift. The electronic nose comprised 18 metal-oxide semiconductor (MOS) sensors (Shanghai Baosheng Industrial Development Co., Ltd., Shanghai, China); each sensor exhibited a characteristic response to different classes of volatile compounds, and the combined sensor array provided a global fingerprint of the sample headspace.

Headspace gas was injected into the sensor chamber at a flow rate of 1.0 L/min. The detection time was 60 s, followed by a 120 s cleaning period to restore the sensor baseline. An automatic zero calibration of 10 s was performed before each measurement [27]. Sensor response signals were recorded and used for subsequent statistical analysis. For data preprocessing, the sensor response signals were first baseline-corrected by subtracting the mean response of the last 10 s of the cleaning period, and the corrected responses were then normalized (to the maximum response across the sensor array) to remove systematic differences in absolute signal intensity before multivariate analysis.

#### 2.3.6. Electronic Tongue Analysis

The taste characteristics were analyzed using an electronic tongue system (SA402B; Insent Inc., Atsugi-shi, Kanagawa, Japan) following the manufacturer’s protocols. The system was equipped with a sensor array comprising five sensors detecting bitterness (SB2), sourness (CA0), saltiness (CT0), umami (AAE), astringency (AE1), and a reference electrode, which allowed the evaluation of bitterness, sourness, saltiness, umami, astringency, richness, and aftertaste.

Prior to measurement, the sensor array was stabilized using standardized cleaning and conditioning procedures to ensure signal reproducibility. The sensors were immersed in the sample solution for 30 s; each sample was measured in triplicate, and the response potentials (mV) relative to the reference solution were recorded automatically.

The sensor array was calibrated daily against the manufacturer’s standard conditioning and reference solutions before measurement to ensure signal reproducibility across samples. For data preprocessing, the raw response potentials were expressed relative to the reference solution, and the data were normalized (Z-score normalization) prior to principal component analysis. After each measurement, the sensors were rinsed and regenerated using the designated cleaning and conditioning solutions following the manufacturer’s protocol to restore sensor performance and minimize carryover. All samples were analyzed under identical conditions, and the sensor response data were used for subsequent statistical and multivariate analyses.

### 2.4. Statistical Analysis

All experimental data were presented as mean ± standard deviation, based on three independent brewing replicates (*n* = 3). Statistical analysis was performed using IBM SPSS Statistics (Version 27.0; IBM Corp., Armonk, NY, USA). The normality of the data distribution was assessed using the Shapiro–Wilk test, and the homogeneity of variance was verified using Levene’s test, prior to all parametric statistical analyses. For single-factor comparisons, differences among samples were evaluated by one-way analysis of variance (ANOVA) followed by Duncan’s multiple-range test. For the carbohydrate structural parameters, a two-way ANOVA was performed with mashing method and fermentation stage as fixed factors, including their interaction; simple main effects were examined where a significant interaction was detected, and effect sizes (partial eta-squared, η^2^_p_) were calculated. A significance level of *p* < 0.05 was used unless otherwise stated. For comparisons involving a single factor, the differences among samples were evaluated using one-way analysis of variance (ANOVA) followed by Duncan’s multiple range test. For carbohydrate structural parameters [low-degree polymerization fraction (LowDP%), average DP (AvgDP), high-degree polymerization fraction (HighDP%), and Mw], a two-way ANOVA was performed, with mashing method and fermentation stage as fixed factors. The main effects of each factor and their interaction (mashing method × fermentation stage) were assessed. When a significant interaction was detected, simple main effects were examined. The assumptions of normality and homogeneity of variance were verified prior to all ANOVA procedures. A significance level of *p* < 0.05 was used for all tests unless otherwise stated. The effect sizes were calculated for two-way ANOVA to indicate the magnitude of each effect.

Principal component analysis (PCA) and orthogonal partial least squares discriminant analysis (OPLS-DA) of electronic nose, electronic tongue, and volatile compound profiles were performed using SIMCA (Version 14.1; Sartorius AG, Göttingen, Germany). Model validity was evaluated using *R*^2^ and *Q*^2^ values.

Data visualization, including fermentation kinetics curves and profiles of organic acids and amino acids, was performed using OriginPro 2021 (OriginLab Corporation, Northampton, MA, USA). Hierarchical clustering heatmaps of volatile compounds were generated using R software (RStudio (2025.09.2+418); Posit Software, Boston, MA, USA).

For OPLS-DA of volatile compound profiles, the model validity was assessed using *R*^2^*X*, *R*^2^*Y*, and *Q*^2^ parameters, with *Q*^2^ obtained by seven-fold cross-validation. The model robustness against overfitting was verified by permutation testing (200 random permutations of the class labels). Differential volatile compounds were selected based on variable importance in projection (VIP) values > 1 derived from the OPLS-DA model, rather than by univariate hypothesis testing across all 126 variables. Therefore, no additional correction for multiple comparisons was required for the VIP-based selection itself. Volatile compounds reported as “not detected” (n.d.) in a given sample—that is, compounds whose target peak was not recognized by mass spectral matching or whose experimental retention index deviated excessively from the literature value—were assigned a value of zero for that sample in the multivariate models. Compounds with a relative standard deviation (RSD) exceeding 50% across replicate samples were flagged as analytically unstable and excluded from multivariate discriminant models.

## 3. Results

### 3.1. Structural Characteristics of Wort Carbohydrates

The DP distribution of carbohydrates differed between the two mashing strategies. In the wort stage, DMM generated numerically higher proportions of low-degree polymerization saccharides (DP1-DP3) than IMM, although no statistically significant difference was observed in the overall LowDP% value between the two worts (Figure 2A and Table 1).

The distribution of carbohydrate fractions also changed during fermentation. From the wort stage to the finished beer stage, both mashing methods exhibited a decrease in the proportion of LowDP%, accompanied by a corresponding increase in HighDP% and AvgDP.

Two-way ANOVA revealed a highly significant effect of the fermentation stage on the structural parameters of carbohydrates (*p* < 0.001). Mashing method also significantly influenced several structural parameters, although the magnitude of the effect varied among parameters. Moreover, a significant interaction between mashing method and fermentation stage was observed (*p* < 0.001), indicating that the structural evolution of carbohydrates during fermentation differed between the two mashing strategies (Table A2).

In the wort stage, DMM wort contained a relatively higher LowDP% (DP1–DP3), whereas the HighDP% (DP4–DP5) was comparable between DMM and IMM worts (Figure 2A). No significant differences were observed in AvgDP, LowDP%, HighDP%, or Mw between the two wort samples. After fermentation, DMM beer exhibited significantly higher AvgDP, HighDP%, and Mw than IMM beer (*p* < 0.05), whereas LowDP% was significantly lower (Table 1). It should be noted that the DP distribution reported here was determined after complete enzymatic debranching, and therefore reflects the chain-length profile of all constituent glucan chains, including those originally present as linear branches on larger dextrin molecules. Consequently, the LowDP% values should be interpreted as an index of the proportion of short glucan chains in the total carbohydrate pool, rather than as a direct measure of the fermentable sugar (glucose, maltose, and maltotriose) concentration in the native (non-debranched) wort. The debranching treatment may overestimate the LowDP% relative to the native state, and hence the observed differences between treatments should be interpreted as relative comparisons rather than absolute compositional characterizations.

### 3.2. Fermentation Performance and Physicochemical Properties of Beers

The physicochemical properties of beers produced using the two mashing strategies are summarized in Table 1. Compared with DMM beer, IMM beer exhibited significantly higher alcohol content, original extract, real extract, color value, and caloric value (*p* < 0.05). In contrast, the real degree of fermentation of DMM beer was significantly higher than that of IMM beer.

The fermentation kinetics of the two worts are shown in Figure 2B. Both worts exhibited a continuous decline in soluble sugar content during fermentation. In IMM wort, the soluble sugar content decreased rapidly in the early fermentation stage and approached a stable level within approximately 36 h. In contrast, DMM wort exhibited a more gradual and prolonged decrease in sugar content, with the decline continuing until approximately 60 h.

VDKs, including diacetyl and 2,3-pentanedione, were quantified in the finished beers. IMM beer exhibited significantly higher concentrations of both diacetyl and 2,3-pentanedione than DMM beer (*p* < 0.05). Similarly, the total potential concentrations of these compounds were markedly higher in IMM beer. All measured VDK concentrations remained well below the regulatory limit specified in GB/T 4928-2026.

### 3.3. Organic Acids in Beer and Amino Acids in Wort

#### 3.3.1. Organic Acids

**Figure 3:**
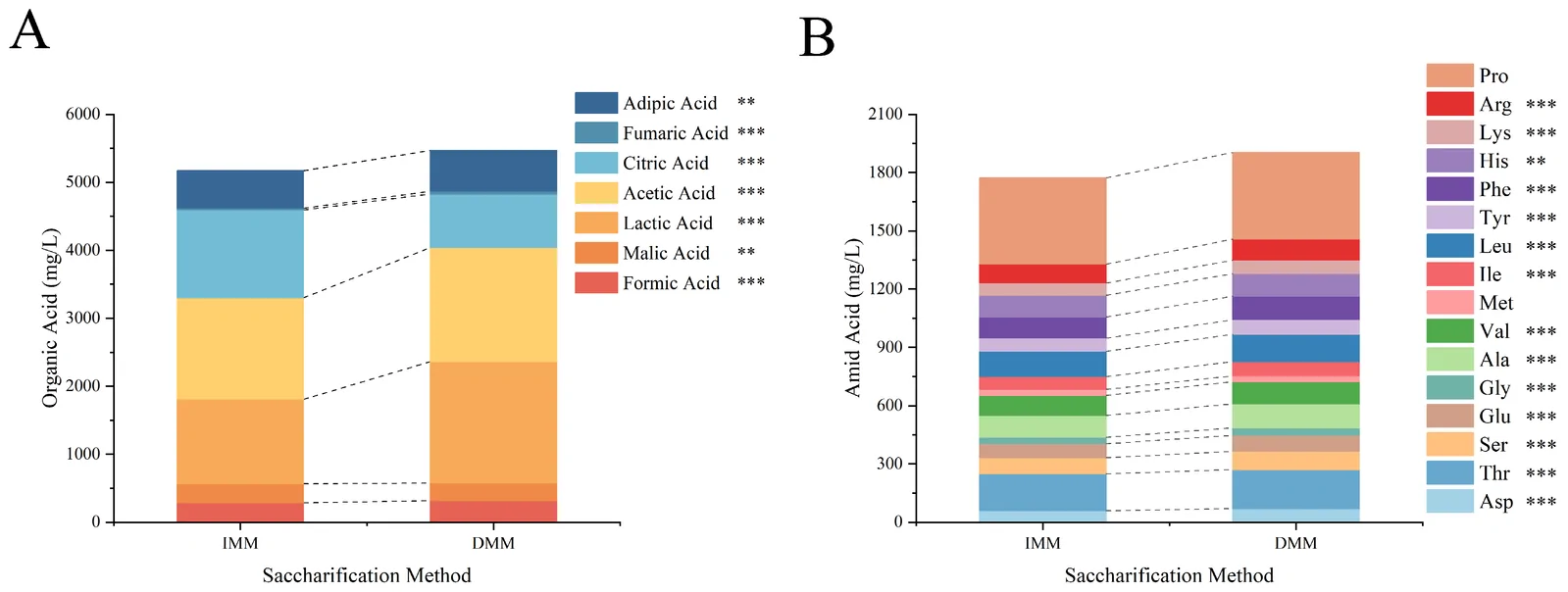
Total organic acids in finished beers (**A**) and total free amino acids in wort (**B**) produced using IMM and DMM. (**), (***) represent a significant difference at *p* < 0.01, *p* < 0.001, respectively.

The concentrations of organic acids in beers produced using the two mashing strategies are shown in Figure 3A. DMM beer exhibited higher concentrations of several organic acids: the concentrations of lactic acid, acetic acid, fumaric acid, formic acid, and adipic acid were significantly higher in DMM beer (*p* < 0.05). In contrast, malic acid and citric acid concentrations were significantly higher in IMM beer. Despite the higher concentrations of several organic acids, the pH of DMM beer was higher than that of IMM beer.

#### 3.3.2. Amino Acids

The amino acid composition of wort samples is presented in Figure 3B. DMM significantly increased the total amino acid concentration in wort compared with IMM. Several amino acids involved in yeast metabolism, including arginine, lysine, histidine, and leucine, were present at significantly higher concentrations in DMM wort. DMM wort also had higher concentrations of serine, glycine, aspartic acid, and glutamic acid, which are amino acids associated with sensory attributes.

### 3.4. Volatile Compounds

Volatile compounds in beers produced using the two mashing strategies were analyzed using HS-SPME-GC-MS. A total of 126 volatile compounds were identified, spanning alcohols, esters, aldehydes, hydrocarbons, ketones, phenols, heterocycles, and other compound classes (Table A1). Of these, 72 compounds were common to both beer samples. Esters and higher alcohols were the dominant volatile groups in both beers, despite obvious differences in volatile composition between IMM and DMM beers.

#### 3.4.1. PCA

**Figure 4:**
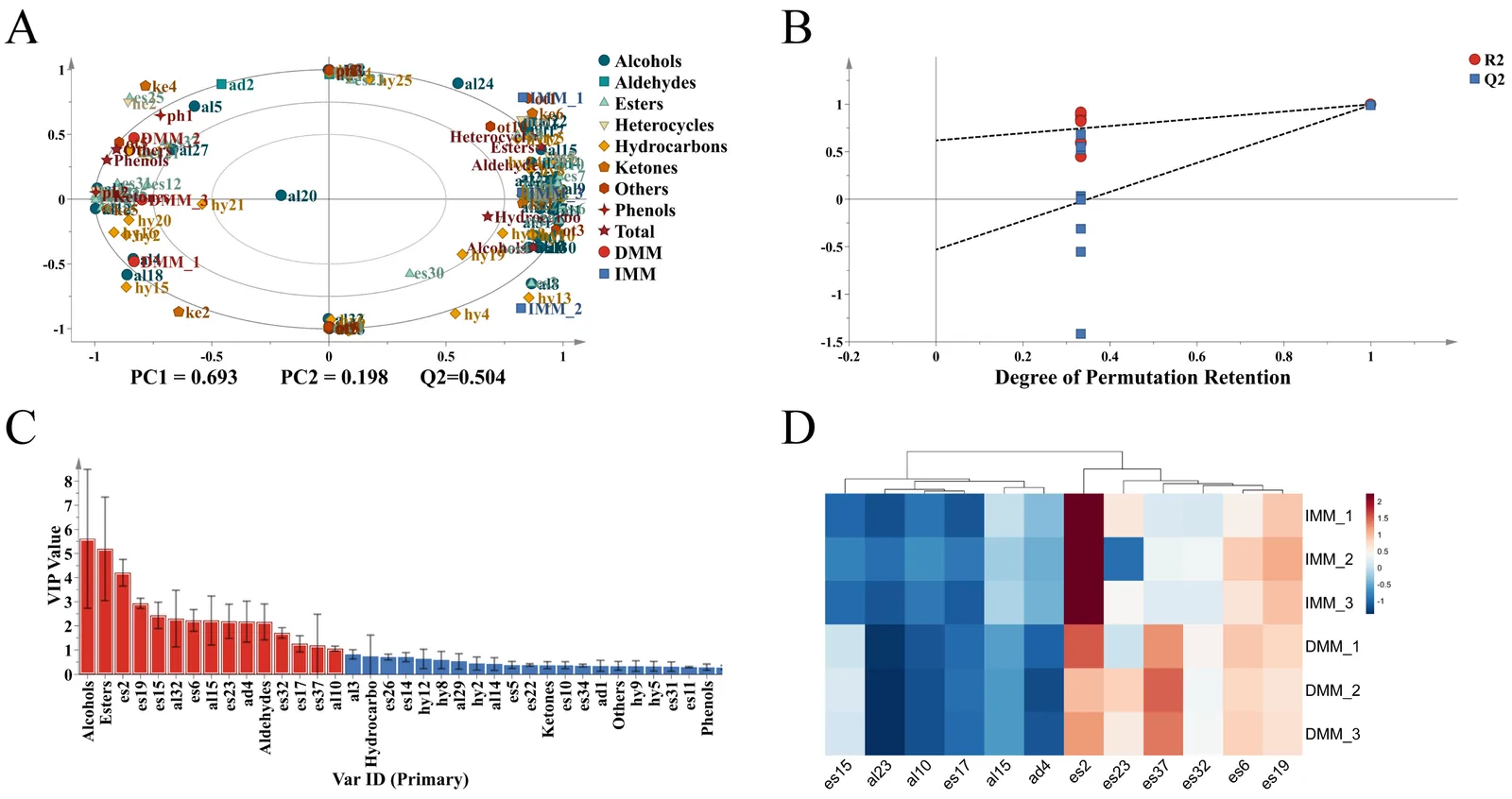
Multivariate statistical analysis of volatile compounds in beers produced using IMM and DMM. (**A**) PCA biplot showing sample scores and compound loading vectors. (**B**) OPLS-DA model validation based on permutation testing. (**C**) VIP score plot identifying key differential compounds. (**D**) Hierarchical clustering heatmap of 12 differential compounds.

PCA was performed using the semi-quantitative concentrations of volatile compounds as variables (Figure 4A). PC1 and PC2 explained 69.3% and 19.8% of the total variance, respectively, together accounting for 89.1% of the overall variability. The PCA biplot showed that IMM and DMM beer samples were separated along PC1, with the compound loading vectors indicating the volatile compounds contributing most strongly to this separation.

#### 3.4.2. OPLS-DA

OPLS-DA was conducted to further identify differential compounds. The model exhibited high explanatory and predictive abilities (*R*^2^*X* = 0.987, *R*^2^*Y* = 0.999, and *Q*^2^ = 0.992). Permutation testing (200 iterations) yielded a *Q*^2^ regression intercept below zero (−0.531, Figure 4B), indicating that the model was robust against overfitting. However, given the limited sample size (*n* = 3 per group) and the large number of predictor variables, the extremely high *R*^2^*Y* and *Q*^2^ values should be interpreted with caution, and the identified discriminant compounds should be regarded as candidates for further validation rather than as definitive biomarkers. Based on the VIP > 1 criterion, 11 volatile compounds were identified as key discriminating compounds (Figure 4C).

#### 3.4.3. Differential Compounds

Based on the VIP > 1 criterion, 11 volatile compounds were identified as key differential compounds. Among these, seven were esters, three were alcohols, and one was an aldehyde (Table 2). One compound, ethyl hexanoate (es23), exhibited a high RSD (≈87%) in IMM samples and was therefore excluded from the discriminant model. The cluster heatmap (Figure 4D) revealed that the medium-chain fatty acid ethyl esters (e.g., ethyl decanoate and ethyl dodecanoate) were more abundant in DMM beer, whereas higher alcohols and aldehydes were relatively more abundant in IMM beer. To assess the potential sensory relevance of the 11 discriminant volatile compounds, we calculated odor activity values (OAVs) by dividing the semi-quantitative concentration of each compound by its reported odor threshold in water (Table 2). Most differential compounds exhibited OAV > 1 in at least one beer, indicating potential aroma activity. Isoamyl acetate (es2) and nonanal (ad4) displayed the highest OAVs in both beers (1956.8 and 1197.3 in IMM, respectively). Among the medium-chain fatty acid ethyl esters, ethyl decanoate (es15) and ethyl dodecanoate (es17) showed higher OAVs in DMM beer (33.5 and 2.9, respectively) than in IMM beer (6.5 and 0.7), whereas acetate esters (isoamyl acetate, 2-phenylethyl acetate, and ethyl acetate) showed higher OAVs in IMM beer.

### 3.5. Instrumental Aroma and Taste Characterization (Electronic Nose and Electronic Tongue)

**Figure 5:**
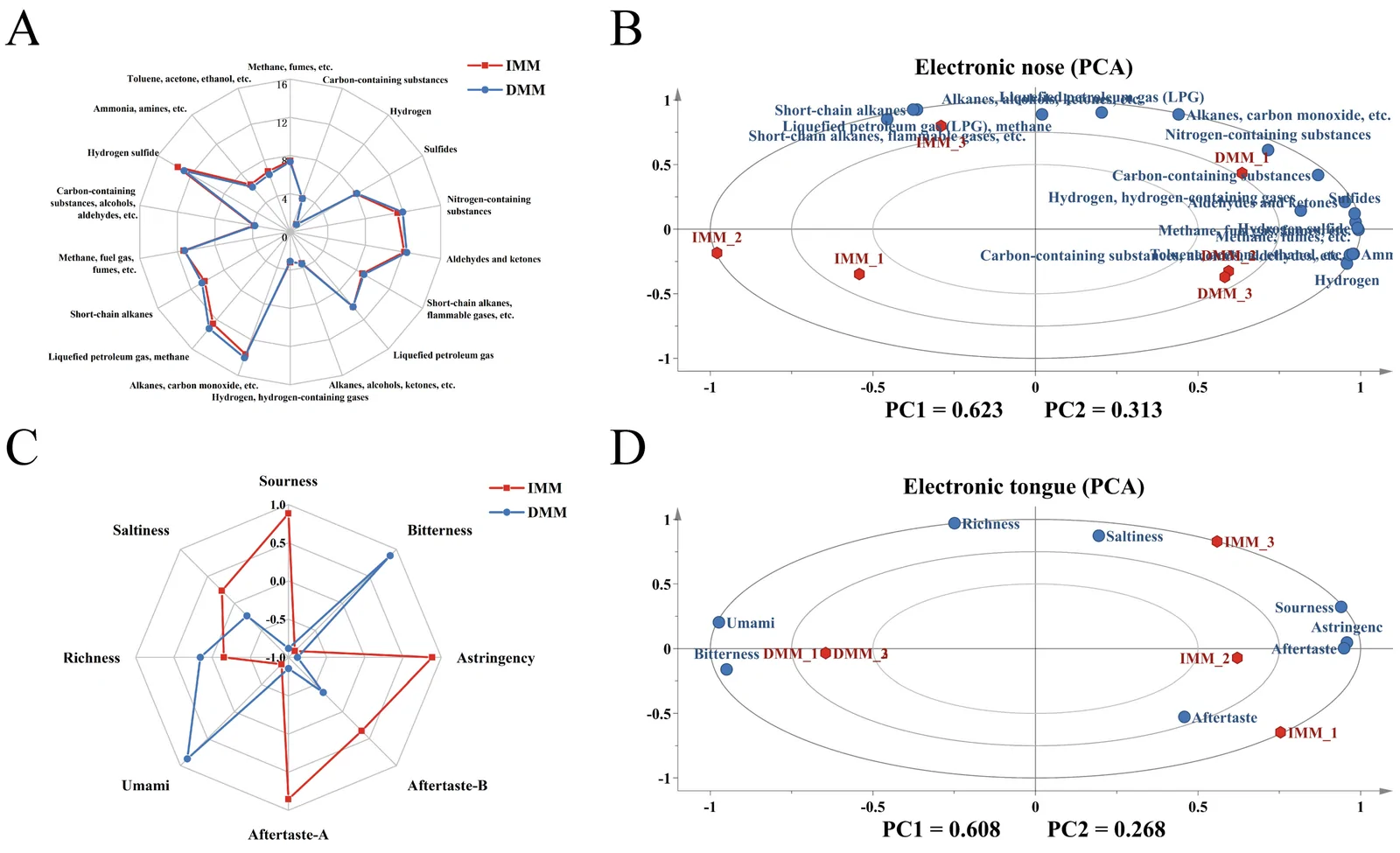
Electronic nose and electronic tongue characterization of beers produced using IMM and DMM. (**A**) Radar plot of electronic nose responses. (**B**) PCA score plot of electronic nose data. (**C**) Radar plot of electronic tongue responses after *Z*-score normalization. (**D**) PCA score plot of normalized electronic tongue data.

The instrumental aroma and taste characteristics of beers produced using the two mashing methods were evaluated by electronic nose and electronic tongue analyses. Radar plots of the electronic nose responses are shown in Figure 5A. Both beers exhibited broadly similar response patterns across the sensor array, despite marked differences in response intensity for several sensors.

PCA was applied to the electronic nose data (Figure 5B). The first two principal components explained 62.3% and 31.3% of the total variance, respectively (cumulative: 93.6%). The score plot separated IMM and DMM beers along PC1. The replicate samples of DMM beer were more tightly clustered than those of IMM beer.

Electronic tongue analysis was performed to evaluate the taste attributes of the beers (Figure 5C). The electronic tongue sensor indicated that DMM beer exhibited higher signal intensities for bitterness, umami, and richness sensors, whereas IMM beer showed relatively higher signal intensities for sourness, saltiness, astringency, and aftertaste sensors. PCA of the electronic tongue data (Figure 5D) gave PC1 and PC2 explaining 60.8% and 26.8% of the variance, respectively (cumulative: 87.6%). The score plot distinguished IMM and DMM beers along PC1. The replicate samples of DMM beer exhibited a more compact distribution in the PCA plot than those of IMM beer.

## 4. Discussion

### 4.1. Influence of Mashing Strategy on the Carbohydrate Structure of Wort

The present study showed differences in the DP distribution between IMM and DMM worts. In the wort stage, DMM wort contained a numerically higher proportion of LowDP% saccharides (DP1–DP3), although this difference did not reach statistical significance. This trend suggests that the decoction step may have promoted the formation of smaller carbohydrate fractions during saccharification.

The higher abundance of LowDP% saccharides in DMM wort may be attributed to the thermal and enzymatic conditions created by the decoction boiling step [9,29]. Boiling a portion of the mash enhances starch gelatinization and improves the accessibility of starch granules to amylolytic enzymes. Gelatinized starch is generally more susceptible to enzymatic hydrolysis than native starch, facilitating the production of smaller oligosaccharides [30]. Moreover, the boiling and return step may shift the balance between α-amylase and β-amylase activities during the subsequent saccharification rests, thereby altering the distribution of fermentable sugars and dextrins in the wort [29]. It should be noted that several methodological factors constrain the interpretation of the DP distribution data. The debranching enzyme treatment used prior to HPAEC-PAD analysis may partially release short linear chains from originally branched dextrins, leading to an overestimation of the LowDP% and a corresponding underestimation of higher-molecular-weight species. Consequently, the absolute values of LowDP% and fermentable sugar content should be interpreted as relative comparisons between treatments rather than as absolute compositional characterizations.

Although DMM wort initially exhibited higher proportions of LowDP% saccharides, the finished DMM beer displayed significantly higher AvgDP and a higher HighDP% compared with IMM beer. This reversal can be explained by the selective utilization of fermentable sugars during fermentation [31]. Brewing yeast preferentially consumes small saccharides—glucose, maltose, and maltotriose—which correspond to the LowDP% carbohydrate. As fermentation proceeds, these fermentable sugars are depleted, resulting in a relative enrichment of higher-molecular-weight dextrins in the residual carbohydrate fraction [32]. The combined effects of mashing-induced carbohydrate distribution and selective sugar consumption during fermentation therefore shape the carbohydrate structure of the finished beer. These findings suggest that the mashing strategy influences not only the initial carbohydrate composition of the wort but also the trajectory of carbohydrate structural evolution throughout fermentation.

### 4.2. Effects of Carbohydrate Composition and Wort Components on Fermentation Behavior

The two mashing strategies yielded distinct carbohydrate profiles, and these differences were reflected in fermentation behavior. Although DMM generated wort with a numerically higher proportion of LowDP% saccharides, IMM wort fermented more rapidly. The soluble sugar content of IMM wort decreased rapidly in the early stage and stabilized within approximately 36 h, whereas DMM fermentation proceeded more gradually for approximately 60 h. This pattern may be related to the differences in the availability and utilization patterns of individual fermentable sugars [17,33,34]. One possible contributing factor is glucose repression. If DMM wort contained more free glucose than maltose—a possibility consistent with its numerically higher DP%—this could transiently suppress the uptake of maltose and maltotriose, thereby slowing the overall fermentation rate [35,36]. However, as individual fermentable sugars were not quantified in real time during fermentation, this interpretation remains speculative. Additionally, the lower original extract of DMM wort may have contributed to the slower attenuation by providing a smaller pool of readily fermentable substrate in absolute terms. Further studies employing real-time sugar-specific monitoring are needed to elucidate the mechanistic basis of the observed differences in fermentation rate.

The differences in fermentation kinetics were also reflected in the physicochemical properties of the final beers. IMM beer exhibited higher ethanol concentration and higher original and real extracts compared with DMM beer, consistent with reports showing that infusion mashing led to faster fermentation and higher alcohol production [7,37]. Although IMM beer had a higher final alcohol content, its RDF was lower than that of DMM beer. This apparent paradox can be explained by the higher original extract in IMM wort: RDF is calculated as the proportion of original extract that has been fermented. Therefore, a higher starting extract implied that even with a larger absolute amount of sugar consumed, the percentage of extract fermented could be lower. The higher original extract remaining in IMM beer also indicated that more residual unfermented material was present. Beyond carbohydrate composition, nitrogen availability and amino acid utilization also influence yeast metabolism during fermentation. Amino acids serve as essential nitrogen sources for yeast growth and are important precursors of fermentation-derived flavor compounds. The higher total amino acid concentration in DMM wort supported sustained yeast metabolic activity, potentially contributing to the prolonged fermentation observed under DMM conditions. Branched-chain amino acids such as valine are directly linked to diacetyl metabolism: Valine uptake suppresses the valine biosynthetic pathway, thereby reducing the production of α-acetolactate, the direct precursor of diacetyl [38]. The lower diacetyl and 2,3-pentanedione concentrations observed in DMM beer may therefore reflect, at least in part, the differences in amino acid availability and uptake kinetics between the two fermentation systems [39]. Although the diacetyl concentration in IMM beer [31.59 μg/L (0.032 mg/L)] was significantly higher than that in DMM beer [11.77 μg/L (0.012 mg/L)], both values remained below the reported sensory detection threshold for diacetyl in beer (0.06 mg/L) [28] and below the limit for premium-grade beer specified in the Chinese national standard GB/T 4927-2025 [40] (≤0.10 mg/L). The observed difference therefore did not imply a flavor defect in either beer but indicated that the mashing strategy quantitatively influenced VDK management. In this study, no separate diacetyl rest was applied; instead, diacetyl reduction occurred during the extended 7-day fermentation at 21 °C, a temperature at which yeast is capable of reducing diacetyl to acetoin and 2,3-butanediol. The lower diacetyl concentration in DMM beer may reflect either a lower production of the precursor α-acetolactate or a more complete utilization and reduction in diacetyl by yeast during the prolonged fermentation, or a combination of both.

These findings indicated that the mashing strategy influenced not only the carbohydrate composition of wort but also yeast fermentation dynamics, amino acid metabolism, and VDK management, collectively contributing to the differences in the physicochemical and flavor characteristics of the finished beers.

An additional observation that merits discussion is the higher color value of IMM beer compared with DMM beer (Table 1). This result appears counterintuitive because the decoction boiling step is generally expected to intensify Maillard reactions and melanoidin formation, thereby increasing color. One possible explanation is that color formation was influenced by factors beyond the mashing boiling step alone. The higher original extract of IMM wort might have contributed to a greater concentration of color precursors, which could undergo Maillard reactions during kettle boiling and throughout fermentation. Additionally, the differences in polyphenol oxidation, pH, and the concentration of color-contributing intermediates might have contributed. Without wort-stage color measurements, the relative contributions of mashing-derived versus boiling-derived color could not be definitively distinguished in this study. Future studies should include color measurements in each processing stage to trace the origin of color differences.

It should be acknowledged that the IMM and DMM worts differed in the original extract (approximately 11.9 °P versus 10.1 °P, respectively, as measured in the finished beers). As identical raw materials and water volumes were used for both treatments, this difference was attributable to the mashing strategy itself. The additional boiling and transfer steps in the decoction process might have reduced the overall extract recovery efficiency. However, the resulting differences in ethanol concentration and osmotic pressure might have partly mediated some of the downstream effects on yeast metabolism and volatile compound formation. The flavor differences observed between IMM and DMM beers therefore reflect the combined influence of differential carbohydrate structure, original gravity, and ethanol concentration, all of which are inherently linked to the mashing strategy. Future studies employing wort standardization to a common original gravity prior to fermentation, or ANCOVA-based statistical correction, may help disentangle the direct effects of carbohydrate structure from those mediated by ethanol concentration.

### 4.3. Influence of Mashing Strategy on the Formation of Volatile Compounds

Distinct volatile profiles were observed between beers produced using the two mashing strategies, suggesting that mashing-induced differences in wort composition were associated with divergent volatile metabolite formation during fermentation. Recent studies have shown that the specific gravity, carbohydrate composition, nitrogen source, and lipid content of wort can significantly alter the concentrations of higher alcohols and esters during fermentation, underscoring the impact of wort composition on final aroma [41]. In the present study, OPLS-DA-based VIP analysis identified esters as the predominant discriminating volatile group between IMM and DMM beers. This finding suggested that mashing strategy modulated ester biosynthesis through its influence on yeast metabolic activity.

Esters, which contribute fruity and floral sensory notes, are formed primarily through the enzymatic condensation of alcohols and acyl-CoA molecules catalyzed by alcohol acetyltransferases in yeast [42,43,44]. Medium-chain fatty acid ethyl esters were enriched in DMM beer, whereas acetate esters were more abundant in IMM beer. This class-specific distribution might reflect differences in fermentation dynamics and precursor availability [18]. Acetate ester formation is catalyzed by alcohol acetyltransferases and depends on the availability of acetyl-CoA and higher alcohols; the faster fermentation and higher alcohol production in IMM might have favored acetate ester synthesis. In contrast, medium-chain fatty acid ethyl esters are formed through the esterification of ethanol with medium-chain fatty acids, a pathway that might be favored under the prolonged metabolic activity observed in DMM fermentation [43,44]. Slower fermentation can prolong yeast metabolic activity and maintain a more balanced flux between alcohol formation and esterification reactions [45]. Furthermore, the variations in carbohydrate composition and sugar utilization patterns may affect intracellular acetyl-CoA availability, a key precursor for ester biosynthesis [44].

Several higher alcohols and aldehydes were more abundant in IMM beer. These compounds can arise from amino acid catabolism via the Ehrlich pathway, lipid oxidation, or intermediate fermentation metabolites. Their elevated concentrations in IMM beer might reflect the combined effects of a higher original extract, which facilitated greater precursor availability for yeast metabolism, and a more vigorous early fermentation, which promoted the rapid uptake and turnover of amino acids through the Ehrlich pathway. The interplay of these two factors, both inherently linked to the infusion mashing process, likely accounted for the higher concentrations of alcohols and aldehydes observed in IMM beer compared with DMM beer. Branched-chain amino acids such as valine and leucine are directly linked to diacetyl formation and reduction, and their catabolism through the Ehrlich pathway generates higher concentrations of alcohols and acetate esters that shape fruity and floral notes in beer [38,46]. Taken together, the observed differences in volatile composition are likely to reflect the combined influence of carbohydrate structure, yeast metabolic activity, and fermentation conditions associated with each mashing strategy [45,46,47]. Compared with prior comparative studies of mashing methods that focused on antioxidant capacity [11,14] or differences in physicochemical properties, the present study provided volatile-resolved discrimination between IMM and DMM beers and identified specific ester subclasses (acetate esters versus medium-chain fatty acid ethyl esters) differentially associated with each mashing strategy. This finding was also consistent with and extended the work of Lin et al. [47], which showed that mashing-driven changes in amino acid availability regulated beer aroma. Lin et al. focused on protease-mediated amino acid release. However, the present study demonstrated that the choice between infusion and decoction mashing with a thermal-strategy difference can likewise reshape the amino acid and volatile profiles of wheat beer, with acetate esters favored under the rapid fermentation of IMM and medium-chain ethyl esters enriched under the prolonged fermentation of DMM. No previous study has reported this class-resolved effect of mashing strategy on ester formation in wheat beer.

These results indicated that mashing strategy not only influenced carbohydrate composition and fermentation behavior but also indirectly modulated the formation of aroma-active compounds in wheat beer. This interplay highlights the importance of upstream processing conditions in shaping the final instrumental aroma profile of fermented beverages.

### 4.4. Implications for Instrumental Aroma Characteristics and Beer Quality

The compositional differences between IMM and DMM beers were further reflected in their instrumentally assessed characteristics. Electronic nose analysis revealed a clear discrimination between the two beer samples, indicating that the differences in volatile compound profiles were associated with distinguishable differences in overall instrumental aroma patterns.

Instrumental taste responses in beer are influenced by both volatile compounds and nonvolatile constituents, including organic acids, amino acids, and residual carbohydrates [48,49]. Electronic tongue analysis indicated differences between the two beers in several sensor responses, particularly those corresponding to bitterness, sourness, and umami. These differences were likely associated with the variations in organic acid composition, amino acid profiles, and dextrin content, all of which influenced the overall balance and mouthfeel of beer [49,50].

Despite higher concentrations of several organic acids (lactic, acetic, fumaric, formic, and adipic acids) in DMM beer, the pH of DMM beer remained higher than that of IMM beer, while total titratable acidity did not differ significantly between the two beers (Table 1). This apparent inconsistency might be explained by the differences in the composition of the beer matrix. IMM beer contained higher concentrations of malic acid and citric acid, which had lower p*K*_a_ values (malic acid: p*K*_a1_ = 3.40, p*K*_a2_ = 5.11; citric acid: p*K*_a1_ = 3.13, p*K*_a2_ = 4.76, p*K*_a3_ = 6.40) and therefore contributed more to free acidity (lower pH) at beer-relevant pH values than the weaker acids that predominated in DMM beer [51,52]. Organic acids, such as lactic, malic, and citric acids, contribute to the perception of acidity and freshness, but their sensory impact depends on concentration, dissociation constants, and interactions with other beer components [52,53,54]. Moreover, the higher content of high-molecular-weight dextrins and non-starch polysaccharides in DMM beer may enhance sweetness and palate fullness while physically modulating aroma and taste compound release. Such macromolecular matrix effects can mask sourness and shift the perceived balance between sweetness and acidity, thereby influencing electronic tongue response patterns. The observed differences in instrumental taste responses between IMM and DMM beers were also consistent with the concept that nonvolatile macromolecules modulate palate-related attributes in beer [55,56]. The higher Mw of residual carbohydrates (higher AvgDP and Mw) in DMM beer may contribute to its higher instrumental richness and bitterness responses, whereas the higher concentrations of small organic acids and acetate esters in IMM beer are consistent with its higher sourness response. By linking mashing-driven differences in the molecular structure of carbohydrates to instrumental taste characteristics, the present study extends these earlier findings from a process-engineering perspective and highlights mashing strategy as a practical lever for modulating the macromolecular and flavor profiles of wheat beer.

These results suggest an association of mashing strategy with beer quality through a series of interrelated biochemical changes. Differences in thermal treatment are reflected in the carbohydrate structure of wort, which is associated with distinct fermentation kinetics, yeast metabolism, and volatile compound profiles. These compositional differences are further reflected in the instrumental aroma and taste characteristics of the finished beer. These findings offer insights for optimizing brewing processes to modulate the instrumental aroma profile of wheat beer.

## 5. Conclusions

This study examined the effects of IMM and DMM on carbohydrate structure, fermentation behavior, and flavor formation in wheat beer. The results indicated that the two mashing strategies produced distinct carbohydrate profiles in wort, and these profiles were associated with differences in yeast fermentation kinetics and final beer composition.

Clear differences in volatile compound profiles were observed between beers produced using the two mashing strategies, with esters identified as the principal discriminating volatile group through multivariate analysis. These findings suggested that mashing-induced differences in carbohydrate structural characteristics were associated with downstream differences in ester biosynthesis during fermentation. Under DMM conditions, the fermentable sugar release and utilization patterns were associated with a volatile profile characterized by higher concentrations of medium-chain fatty acid ethyl esters, whereas the relatively rapid attenuation under IMM conditions was accompanied by higher concentrations of several higher alcohols and aldehydes. Taken together, these results indicate associations among mashing strategy, wort carbohydrate structure, yeast metabolic dynamics, and volatile compound profiles in wheat beer brewing.

This study provides new evidence on the associations between mashing strategy, carbohydrate composition, fermentation behavior, and volatile compound profiles in wheat beer. By integrating physicochemical analysis, metabolite profiling, and multivariate instrumental aroma analysis, the study offers a more integrated perspective on the association between mashing processes and beer flavor quality. These findings may inform the optimization of brewing processes aimed at modulating fermentation performance and the flavor profile of wheat beer.

## Figures and Tables

**Table 1:** Physicochemical properties of beers produced using different mashing strategies.

Test Characteristics	IMM Beer	DMM Beer	IMM Wort	DMM Wort
Structural characteristics of carbohydrates				
AvgDP	2.3626 ± 0.0174 ^b^	2.6532 ± 0.0554 ^a^	1.9479 ± 0.0244 ^c^	1.9791 ± 0.0095 ^c^
LowDP%	87.67 ± 0.29 ^b^	76.64 ± 1.38 ^c^	96.74 ± 0.23 ^a^	97.97 ± 0.13 ^a^
HighDP%	12.33 ± 0.29 ^b^	23.36 ± 1.38 ^a^	3.26 ± 0.23 ^c^	2.03 ± 0.13 ^c^
Mw (g/mol)	1682 ± 94 ^b^	2088 ± 135 ^a^	776 ± 36.7 ^c^	673 ± 34.4 ^c^
Physicochemical properties of beer				
Alcohol (% *v*/*v*)	5.01 ± 0.13 ^a^	4.37 ± 0.09 ^b^	-	-
Original extract (% *w*/*w*)	11.9 ± 0.17 ^a^	10.1 ± 0.15 ^b^	-	-
Er (real extract) (% *w*/*w*)	4.28 ± 0.12 ^a^	3.38 ± 0.16 ^b^	-	-
RDF (real degree of fermentation) (%)	65.37 ± 0.13 ^b^	68.04 ± 0.09 ^a^	-	-
pH	4.01 ± 0.07 ^b^	4.3 ± 0.06 ^a^	-	-
Total acid (mL/100 mL)	1.86 ± 0.11 ^a^	1.8 ± 0.09 ^a^	-	-
Vicinal diketones				
Diacetyl (μg/L)	31.59 ± 1.46 ^a^	11.77 ± 0.74 ^b^	-	-
2,3-Pentanedione (μg/L)	14.75 ± 1.16 ^a^	3.76 ± 0.29 ^b^	-	-
Total diacetyl (μg/L)	44.3 ± 1.57 ^a^	23.64 ± 0.69 ^b^	-	-
Total 2,3-pentanedione (μg/L)	16.15 ± 1.6 ^a^	6.96 ± 0.87 ^b^	-	-

All values are expressed as mean ± standard deviation (*n* = 3). Values with different letters(“a”, “b” and “c”) in the same row were significantly different (*p* < 0.05).

**Table 2:** Differential volatile compounds in IMM and DMM beers.

Code	Compounds ^a^	CAS	Aroma ^b^	Threshold (mg/L) ^c^	OAV		Relative Abundance (4-FB eq. (μg/L)) ^d^	
					IMM	DMM	IMM	DMM
al10	1-Octanol	111-87-5	Burned, chemical, metal, moss, mushroom, nut, oil, and soap	0.875	1.46	0.75	1.281 ± 0.0388	0.66 ± 0.0117
al15	1-Propanol, 2-methyl-	78-83-1	Bitter, solvent, and wine	2.3	2.15	0.95	4.94 ± 1.1192	2.177 ± 0.1092
al23	2-Undecanol	1653-30-1	Flower, grass, and nut	0.0086	n.d. ^e^	0.93	n.d. ^e^	0.008 ± 0.0006
ad4	Nonanal	124-19-6	Citrus, cucumber, fat, flower, green, lemon, lime, orange, and tallow	0.0028	1197.5	251.07	3.353 ± 0.8711	0.703 ± 0.1117
es2	1-Butanol, 3-methyl-, acetate	123-92-2	Apple, banana, fruit, honey, pear, and sweet	0.0087	1956.78	829.2	17.024 ± 0.609	7.214 ± 0.3355
es6	Acetic acid, 2-phenylethyl ester	103-45-7	Balsam, flower, fruit, honey, rose, tobacco, tropical, and wine	0.48	18.71	12.9	8.979 ± 0.3427	6.19 ± 0.2073
es15	Decanoic acid, ethyl ester	110-38-3	Brandy, fat, fruit, grape, oil, pear, and wine	0.122	6.51	33.49	0.794 ± 0.0066	4.086 ± 0.4017
es17	Dodecanoic acid, ethyl ester	106-33-2	Fat, flower, leaf, oil, and wax	0.4	0.71	2.91	0.285 ± 0.0417	1.166 ± 0.1334
es19	Ethyl acetate	141-78-6	Acid, ether, fruit, grape, green, pineapple, solvent, sweet, and wine	5	2.12	1.16	10.601 ± 0.053	5.813 ± 0.1354
es32	Octanoic acid, ethyl ester	106-32-1	Apricot, brandy, fat, flower, fruit, pineapple, soap, and wine	0.194	34.2	25.76	6.635 ± 0.063	4.997 ± 0.1603
es37	Propanoic acid, ethyl ester	105-37-3	Apple, banana, fruit, grape, pineapple, rum, and strawberry	1.9	3.53	4	6.698 ± 0.1039	7.603 ± 0.7902

^a^ Volatile flavor compounds detected in the beer. ^b^ Aroma descriptor was derived from the web pages: https://www.flavornet.org/flavornet.html (accessed on 8 May 2026). ^c^ Odor threshold from the literature [28]. ^d^ Values are expressed as semi-quantitative concentrations in 4-fluorobenzaldehyde equivalents (μg/L), calculated as (peak area of compound/peak area of internal standard) × concentration of the internal standard. ^e^ “n.d.” (not detected) indicates that the compound was not detected in that sample because its target peak was not recognized by mass spectral matching or its experimental retention index deviated excessively from the literature value; n.d. values were treated as zero in the multivariate statistical models.

## Data Availability

The data supporting the findings of this study are available from the corresponding author upon reasonable request.

## Abbreviations

DMM	Decoction mashing method
DMSO	Dimethyl sulfoxide
GPC	Gel permeation chromatography
HPAEC-PAD	High-performance anion-exchange chromatography with pulsed amperometric detection
HPLC	High-performance liquid chromatography
HS-SPME-GC-MS	Headspace solid-phase microextraction coupled with gas chromatography–mass spectrometry
IMM	Infusion mashing method
VDK	Vicinal diketone

## Appendix A

**Table A1:** Volatile flavor compounds in beer produced using different mashing strategies (*n* = 3).

Code	Compounds ^a^	CAS	Aroma ^b^	Lib. RI	Similarity	Reverse	Probability	Relative Abundance (4-FB eq. (μg/L)) ^c^	
								IMM	DMM
	Alcohols								
al1	1,6,10-Dodecatrien-3-ol, 3,7,11-trimethyl-	7212-44-4	Bark, flower, green, leaf, and wood	2033 ± 14 (71)	909	909	33.9	0.097 ± 0.0063	0.062 ± 0.0054
al2	1-Butanol	71-36-3	Alcohol, banana, fruit, fusel, medicine, and wine	1142 ± 11 (292)	953	953	73.9	0.057 ± 0.006	0.042 ± 0.002
al3	1-Decanol	112-30-1	Fat, flower, lemon, and orange	1760 ± 9 (70)	960	963	32.6	0.275 ± 0.0029	0.645 ± 0.0489
al4	1-Dodecanol	112-53-8	Earth, flower, soap, and violet	1966 ± 10 (84)	920	954	37.9	n.d.	0.062 ± 0.0024
al5	1-Heptanol	111-70-6	Chemical, fruit, green, herb, and oil	1453 ± 8 (127)	971	971	66.8	0.064 ± 0.0059	0.073 ± 0.0099
al6	1-Hexanol	111-27-3	Alcohol, banana, flower, fruit, grass, green, herb, oil, and wine	1355 ± 7 (347)	956	956	70.3	0.13 ± 0.013	0.1 ± 0.0003
al7	1-Hexanol, 2-ethyl-	104-76-7	Citrus, fresh, flower, green, oil, and rose	1491 ± 5 (154)	932	932	70	0.043 ± 0.0009	0.04 ± 0.0011
al8	1-Hexanol, 3-methyl-	13231-81-7	n.f.	1413 ± 0 (3)	892	892	52	n.d.	0.005 ± 0.0004
al9	1-Nonanol	143-08-8	Citrus, fat, flower, green, lemon, oil, orange, and rose	1660 ± 7 (95)	930	930	56.2	0.036 ± 0.0002	0.023 ± 0.0023
al10	1-Octanol	111-87-5	Burned, chemical, metal, moss, mushroom, nut, oil, and soap	1557 ± 8 (338)	965	965	59.9	1.281 ± 0.0388	0.66 ± 0.0117
al11	1-Octen-3-ol	3391-86-4	Chemical, earth, grass, mushroom, and oil	1450 ± 7 (311)	893	893	80.4	0.025 ± 0.0015	n. d
al12	1-Pentanol	71-41-0	Balsam, fruit, green, nut, and yeast	1250 ± 9 (279)	937	937	81.7	0.012 ± 0.0001	0.015 ± 0.0002
al13	1-Pentanol, 4-methyl-	626-89-1	Cheese and green	1315 ± 13 (46)	956	956	80.7	0.029 ± 0.0022	0.017 ± 0.0016
al14	1-Propanol	71-23-8	Alcohol, fruit, and must	1036 ± 9 (143)	936	942	97	0.796 ± 0.0293	0.684 ± 0.0536
al15	1-Propanol, 2-methyl-	78-83-1	Bitter, solvent, and wine	1092 ± 9 (269)	957	957	86.8	4.94 ± 1.1192	2.177 ± 0.1092
al16	1-Propanol, 3-(methylthio)-	505-10-2	Bean, onion, potato, soup, sulfur, and vegetable	1719 ± 9 (91)	959	959	96.9	n.d.	0.046 ± 0.0014
al17	1-Propanol, 3-ethoxy-	111-35-3	Fruit	1373 ± 9 (24)	937	942	96.7	0.049 ± 0.0063	0.036 ± 0.0039
al18	1-Tetradecanol	112-72-1	Wax	2165 ± 10 (27)	930	930	10.7	n.d.	0.009 ± 0.0001
al19	2,6,10-Dodecatrien-1-ol, 3,7,11-trimethyl-	4602-84-0	Flower and perfume	2350 ± 8 (30)	882	902	24.8	0.083 ± 0.0112	0.061 ± 0.0025
al20	2,6-Octadien-1-ol, 3,7-dimethyl-, (*Z*)-	106-25-2	Citrus, flower, lemon, lime, rose, and seaweed	1797 ± 11 (229)	895	895	47.4	0.094 ± 0.0049	0.097 ± 0.0091
al21	2-Furanmethanol	98-00-0	Burned, coffee, and meat	1660 ± 9 (154)	898	904	83.5	n.d.	0.052 ± 0.0146
al22	2-Octanol	123-96-6	Cheese, cucumber, fat, green, mushroom, nut, oil, and soap	1412 ± 12 (30)	854	854	38.2	n.d.	0.005 ± 0.0009
al23	2-Undecanol	1653-30-1	Flower, grass, and nut	1717 ± 6 (26)	853	853	35.5	n.d.	0.008 ± 0.0006
al24	3-Buten-1-ol, 3-methyl-	763-32-6	Fresh and fruit	1248 ± 8(72)	900	907	96.9	0.054 ± 0.0368	n.d.
al25	4-Penten-1-ol	821-09-0	n.f.	1299 ± 6 (15)	927	927	95	0.016 ± 0.0024	0.004 ± 0.0003
al26	6-Octen-1-ol, 7-methyl-3-methylene-	13066-51-8	n.f.	1800 ± 0 (1)	876	890	80.7	0.006 ± 0.0002	0.004 ± 0.0002
al27	Anethole	104-46-1	Anise, herb, spice, and sweet	1817 ± 5 (9)	884	884	35.6	0.004 ± 0.002	0.036 ± 0.0302
al28	Benzothiazole	95-16-9	Coffee, green, meat, and rubber	1958 ± 12 (53)	831	878	95.2	n.d.	0.008 ± 0.0014
al29	Butylated hydroxytoluene	128-37-0	n.f.	1909 ± 7 (37)	885	898	80.6	0.365 ± 0.0086	n.d.
al30	Eucalyptol	470-82-6	Camphor, cool, herb, leaf, mint, pine, spice	1213 ± 9 (356)	913	913	85.5	0.03 ± 0.0063	0.015 ± 0.0001
al31	Indole	120-72-9	Animal, feces, flower, and mothball	2445 ± 10 (46)	893	893	75.6	0.003 ± 0.0002	0.001 ± 0.00001
al32	Phenylethyl alcohol	60-12-8	n.f.	1906 ± 15 (423)	901	901	50.8	14.219 ± 9.5674	n.d.
al33	α-Terpineol	98-55-5	Anise, flower, lemon, lilac, mint, oil, pine, and wood	1697 ± 10 (594)	875	909	59	0.021 ± 0.0001	0.017 ± 0.0012
Alcohols	Total							22.765 ± 8.539	5.046 ± 0.2422
	Aldehydes								
ad1	Benzaldehyde	100-52-7	Almond, burned, cherry, fruit, malt, and sugar	1520 ± 14 (471)	940	948	86.4	0.215 ± 0.004	n.d.
ad2	Benzaldehyde, 3,4-dimethyl-	5973-71-7	n.f.	1790 ± 0 (1)	942	952	42.6	n.d.	0.322 ± 0.063
ad3	Hexanal	66-25-1	Apple, fat, grass, green, herb, leaf, plum, and tallow	1083 ± 8 (553)	815	838	74.1	0.012 ± 0.0079	n.d.
ad4	Nonanal	124-19-6	Citrus, cucumber, fat, flower, green, lemon, lime, orange, and tallow	1391 ± 8 (461)	953	953	83.4	3.353 ± 0.8711	0.703 ± 0.1117
Aldehydes	Total							3.666 ± 0.7468	1.06 ± 0.0146
	Hydrocarbons								
hy1	Dodecane	112-40-3	Alkane and fusel	1200	941	941	27.6	0.025 ± 0.0052	n.d.
hy2	Hexadecane	544-76-3	n.f.	1600	946	953	17.2	0.019 ± 0.0069	n.d.
hy3	Tridecane	629-50-5	n.f.	1300	916	922	20.9	n.d.	0.054 ± 0.0212
hy4	Tridecane, 3-methyl-	6418-41-3	n.f.	1366 ± 1 (2)	915	921	35.8	0.006 ± 0.0034	n.d.
hy5	Undecane	1120-21-4	n.f.	1100	936	936	42.8	0.106 ± 0.0105	n.d.
hy6	(3*E*,5*Z*)-1,3,5-Undecatriene	51447-08-6	Citrus, flower, green, herb, lavender, lime, pine, and wood	1387 ± 5 (11)	871	871	48.2	n.d.	0.048 ± 0.0378
hy7	1,3-Cyclopentadiene	542-92-7	n.f.	740 ± 5 (2)	902	909	61.8	n.d.	0.552 ± 0.0567
hy8	Bicyclo [4.2.0] octa-1,3,5-triene	694-87-1	n.f.	1269 ± 3 (5)	956	962	47.2	0.644 ± 0.0211	n.d.
hy9	Limonene	138-86-3	Citrus, lemon, mint, and orange	1200 ± 7 (759)	880	917	43.6	0.112 ± 0.004	n.d.
hy10	1H-Indene, 2,3-dihydro-4-methyl-	824-22-6	n.f.	1468 ± 5 (4)	906	906	29.2	0.021 ± 0.0003	n.d.
hy11	Benzene, (dichloromethyl)-	98-87-3	n.f.	1672 ± 0 (3)	915	925	60	0.014 ± 0.0008	0.004 ± 0.0001
hy12	Benzene, 1,2,4,5-tetramethyl-	95-93-2	n.f.	1433 ± 16 (21)	947	947	25.9	0.287 ± 0.1447	0.059 ± 0.0144
hy13	Benzene, 1,3-dimethyl-	108-38-3	Plastic	1143 ± 10 (154)	919	924	31.2	n.d.	0.009 ± 0.006
hy14	Benzene, 1-chloro-3-methyl-	108-41-8	n.f.	1291 ± 3 (5)	881	904	48.5	0.013 ± 0.0068	n.d.
hy15	Benzene, 1-ethyl-2,3-dimethyl-	933-98-2	n.f.	1369 ± 6 (12)	911	924	33.9	n.d.	0.009 ± 0.0001
hy16	Benzene, 1-ethyl-2,4,5-trimethyl-	17851-27-3	n.f.	1391 ± 0 (3)	851	863	37.2	0.001 ± 0.0005	0.008 ± 0.0026
hy17	Benzene, 1-ethyl-2,4-dimethyl-	874-41-9	n.f.	1348 ± 4 (12)	922	922	15.8	0.009 ± 0.0064	0.021 ± 0.0012
hy18	Benzene, 1-ethyl-3,5-dimethyl-	934-74-7	n.f.	1319 ± 6 (14)	917	917	22.8	n.d.	0.008 ± 0.006
hy19	Benzene, 1-methyl-3-(1-methylethyl)-	535-77-3	Carrot, earth, herb, medicine, spice, and wood	1269 ± 9 (35)	907	907	19.1	0.031 ± 0.0218	n.d.
hy20	Benzene, 1-methyl-3-propyl-	1074-43-7	Chemical	1297 ± 18 (17)	899	899	67.1	0.007 ± 0.0036	n.d.
hy21	Benzene, 2-ethyl-1,4-dimethyl-	1758-88-9	n.f.	1340 ± 16 (16)	891	891	19.7	n.d.	0.009 ± 0.0058
hy22	Benzene, 4-ethenyl-1,2-dimethoxy-	6380-23-0	n.f.	2027 ± 13 (4)	880	880	77	0.009 ± 0.0007	n.d.
hy23	Benzene, pentamethyl-	700-12-9	n.f.	1656 ± 11 (2)	887	887	74	0.001 ± 0.0001	n.d.
hy24	Naphthalene	91-20-3	Dry, mothball, and tar	1745 ± 18 (93)	967	967	58.6	0.063 ± 0.0034	0.051 ± 0.0004
hy25	o-Xylene	95-47-6	Geranium	1186 ± 8 (132)	926	933	31.1	0.023 ± 0.0198	n.d.
Hydrocarbons	Total							1.423 ± 0.1094	1.039 ± 0.3416
	Esters								
es1	1,3-Propanediol, diacetate	628-66-0	n.f.	1655 ± 5 (3)	931	943	97.3	0.01 ± 0.0003	n.d.
es2	1-Butanol, 3-methyl-, acetate	123-92-2	Apple, banana, fruit, honey, pear, and sweet	1122 ± 7 (168)	954	954	75.9	17.024 ± 0.609	7.214 ± 0.3355
es3	1-Butanol, 3-methyl-, propanoate	105-68-0	Apricot, banana, fruit, pineapple, and sweet	1185 ± 7 (16)	939	944	74.6	n.d.	0.086 ± 0.0005
es4	2,6,10-Dodecatrien-1-ol, 3,7,11-trimethyl-, acetate, (*E*,*E*)-	4128-17-0	n.f.	2260 ± 15 (16)	858	858	42.5	0.029 ± 0.0068	0.019 ± 0.003
es5	2-Propenoic acid, butyl ester	141-32-2	n.f.	1193 ± 4 (8)	963	963	91.3	0.095 ± 0.0257	0.012 ± 0.0057
es6	Acetic acid, 2-phenylethyl ester	103-45-7	Balsam, flower, fruit, honey, rose, tobacco, tropical, and wine	1813 ± 15 (142)	951	951	85.2	8.979 ± 0.3427	6.19 ± 0.2073
es7	Acetic acid, butyl ester	123-86-4	Banana, ether, fruit, and solvent	1074 ± 8 (131)	947	947	94.9	0.043 ± 0.0054	0.007 ± 0.0017
es8	Acetic acid, decyl ester	112-17-4	Citrus, fruit, herb, jasmine, lemon, orange, rose, and wood	1680 ± 10 (38)	896	896	31	0.004 ± 0.0003	n.d.
es9	Acetic acid, heptyl ester	112-06-1	Apricot, flower, fruit, rose, and wood	1377 ± 8 (27)	882	882	59.8	0.059 ± 0.0289	0.014 ± 0.0007
es10	Acetic acid, hexyl ester	142-92-7	Apple, banana, flower, fruit, grass, green, herb, pear, and sweet	1272 ± 7 (190)	981	981	95.6	0.144 ± 0.024	0.065 ± 0.0031
es11	Acetic acid, octyl ester	112-14-1	Fruit, green, and oil	1475 ± 8 (67)	930	930	70.2	0.068 ± 0.0007	0.016 ± 0.0001
es12	Acetic acid, pentyl ester	628-63-7	Apple, banana, ether, fruit, and sweet	1176 ± 7 (72)	875	875	88.2	0.003 ± 0.0006	n.d.
es13	Benzeneacetic acid, ethyl ester	101-97-3	Flower, fruit, honey, spice, and sweet	1783 ± 10 (50)	956	956	78.5	0.013 ± 0.0001	0.009 ± 0.0004
es14	Butanoic acid, ethyl ester	105-54-4	Acid, apple, cheese, fruit, gum, pineapple, strawberry, and sweet	1035 ± 8 (251)	962	962	95.8	0.594 ± 0.0477	0.318 ± 0.023
es15	Decanoic acid, ethyl ester	110-38-3	Brandy, fat, fruit, grape, oil, pear, and wine	1638 ± 9 (131)	951	951	78.8	0.794 ± 0.0066	4.086 ± 0.4017
es16	Decanoic acid, methyl ester	110-42-9	Flower, honey, and wine	1593 ± 9 (53)	947	947	65.3	0.008 ± 0.0001	0.052 ± 0.0016
es17	Dodecanoic acid, ethyl ester	106-33-2	Fat, flower, leaf, oil, and wax	1841 ± 9 (84)	953	955	76.5	0.285 ± 0.0417	1.166 ± 0.1334
es18	Dodecanoic acid, methyl ester	111-82-0	Fat, flower, and wine	1804 ± 7 (40)	820	820	79.8	n.d.	0.003 ± 0.0001
es19	Ethyl acetate	141-78-6	Acid, ether, fruit, grape, green, pineapple, solvent, sweet, and wine	888 ± 8 (234)	981	981	95.8	10.601 ± 0.053	5.813 ± 0.1354
es20	Heptanoic acid, ethyl ester	106-30-9	Apricot, cherry, fruit, grape, pineapple, raspberry, strawberry, and wine	1331 ± 8 (55)	929	932	94.8	n.d.	0.024 ± 0.0001
es21	Hexadecanoic acid, ethyl ester	628-97-7	Wax	2251 ± 9 (77)	930	930	63.2	n.d.	0.057 ± 0.013
es22	Hexanoic acid, 2-phenylethyl ester	6290-37-5	Wax	2162 ± 4 (10)	932	932	35.3	0.102 ± 0.0047	0.019 ± 0.0007
es23	Hexanoic acid, ethyl ester	123-66-0	Apple, banana, brandy, fruit, green, pear, pineapple, strawberry, sweet, and wine	1233 ± 9 (275)	977	977	96.3	5.758 ± 5.0131	5.21 ± 1.4652
es24	Hexanoic acid, propyl ester	626-77-7	Fruit and wine	1316 ± 9 (22)	878	878	90	n.d.	0.003 ± 0.0002
es25	Isoamyl laurate	6309-51-9	n.f.	2062 ± 5 (8)	917	917	73.5	n.d.	0.009 ± 0.001
es26	Isobutyl acetate	110-19-0	Apple, banana, currant, ether, fruit, pear, and sweet	1012 ± 8 (89)	956	956	93.1	0.578 ± 0.0279	0.299 ± 0.0123
es27	n-Caprylic acid isobutyl ester	5461-06-3	Fruit and sweet	1548 ± 4 (10)	868	868	90.6	0.003 ± 0.0001	n.d.
es28	Nonanoic acid, ethyl ester	123-29-5	Brandy, fat, fruit, grape, rose, and wine	1531 ± 7 (45)	873	878	85.2	0 ± 0.0002	0.003 ± 0.0002
es29	n-Propyl acetate	109-60-4	Apple, celery, fruit, pear, and raspberry	973 ± 11 (75)	964	973	96.4	0.115 ± 0.0052	0.069 ± 0.0047
es30	Octanoic acid, 2-phenylethyl ester	5457-70-5	n.f.	2355 ± 19 (4)	933	933	65.6	0.01 ± 0.003	0.009 ± 0.0011
es31	Octanoic acid, 3-methylbutyl ester	2035-99-6	n.f.	1658 ± 7 (28)	948	948	79.4	0.063 ± 0.006	0.123 ± 0.0235
es32	Octanoic acid, ethyl ester	106-32-1	Apricot, brandy, fat, flower, fruit, pineapple, soap, and wine	1435 ± 6 (207)	946	946	88.2	6.635 ± 0.063	4.997 ± 0.1603
es33	Octanoic acid, methyl ester	111-11-5	Fruit, green, orange, and wine	1385 ± 7 (61)	962	962	89.7	0.01 ± 0.0024	0.03 ± 0.0048
es34	Pentadecanoic acid, 3-methylbutyl ester	2306-91-4	n.f.	1863 ± 5 (15)	953	953	94.7	0.013 ± 0.0023	0.085 ± 0.0038
es35	Pentanoic acid, ethyl ester	539-82-2	Apple, fruit, gum, pineapple, and yeast	1134 ± 7 (66)	915	925	95.4	0.033 ± 0.0104	0.081 ± 0.0141
es36	Propanoic acid, butyl ester	590-01-2	Apple, apricot, banana, fruit, marzipan, pear, pineapple, and plum	1139 ± 9 (29)	938	938	85.1	0.009 ± 0.0024	n.d.
es37	Propanoic acid, ethyl ester	105-37-3	Apple, banana, fruit, grape, pineapple, rum, and strawberry	953 ± 7 (87)	942	942	96.8	6.698 ± 0.1039	7.603 ± 0.7902
es38	Tetradecanoic acid, ethyl ester	124-06-1	Honey, oil, violet, wax, and wine	2049 ± 11 (59)	949	949	69.7	0.027 ± 0.0024	0.042 ± 0.0022
es39	β-Phenylethyl butyrate	103-52-6	Flower, fruit, honey, rose, sweet, tobacco, and wine	1958 ± 17 (9)	899	899	51.9	0.009 ± 0.001	n.d.
Esters	Total							58.856 ± 5.39	43.733 ± 2.933
	Heterocycles								
he1	Benzofuran, 2,3-dihydro-	496-16-2	n.f.	2389 ± 0 (3)	860	868	67	0.012 ± 0.001	0.028 ± 0.0002
he2	Pyrazine, 3-ethyl-2,5-dimethyl-	13360-65-1	Coffee, earth, nut, potato, and roast	1443 ± 8 (75)	923	934	79.7	n.d.	0.003 ± 0.0001
he3	Pyrazine, tetramethyl-	1124-11-4	Coffee, fermentation, green, and natto	1469 ± 7 (49)	861	906	98.1	0.014 ± 0.0051	n.d.
he4	2(3H)-Furanone, 5-ethyldihydro-	695-06-7	Herb, sweet, and tobacco	1694 ± 16 (72)	927	927	92.1	0.007 ± 0.00001	0.004 ± 0.0002
he5	2(3H)-Furanone, 5-hexyldihydro-	706-14-9	Fat, flower, fruit, peach, and sweet	2138 ± 13 (94)	937	952	77.8	0.003 ± 0.0002	n.d.
he6	2(3H)-Furanone, dihydro-5-pentyl-	104-61-0	Coconut, fat, fruit, peach, and sweet	2024 ± 15 (104)	953	953	72.4	0.064 ± 0.0049	0.044 ± 0.004
Heterocycles	Total							0.101 ± 0.0104	0.079 ± 0.0034
	Ketones								
ke1	2-Heptanone	110-43-0	Banana, cheese, ether, fruit, green, nut, soap, and spice	1182 ± 8 (180)	919	919	83.1	0.009 ± 0.0012	n.d.
ke2	2-Octanone	111-13-7	Cheese, earth, fruit, ketone, mushroom, and soap	1287 ± 8 (70)	883	883	71	0.001 ± 0.0001	n.d.
ke3	Acetone	67-64-1	Apple, ether, fruit, glue, and solvent	819 ± 6 (113)	929	929	82	n.d.	0.014 ± 0.0027
ke4	Acetophenone	98-86-2	Almond, flower, fruit, honey, nut, and water	1647 ± 13 (126)	917	929	58.6	n.d.	0.08 ± 0.013
ke5	Ethanone, 1-(1H-pyrrol-2-yl)-	1072-83-9	Bread, caramel, and walnut	1973 ± 12 (56)	920	920	75.9	0.051 ± 0.0024	0.062 ± 0.0024
ke6	Ethanone, 1-(2-furanyl)-	1192-62-7	Balsam, burned, cocoa, coffee, nut, and sweet	1499 ± 10 (133)	909	920	92	n.d.	0.007 ± 0.0002
Ketones	Total							0.082 ± 0.0185	0.164 ± 0.0137
	Phenols								
ph1	2,4-Di-*tert*-butylphenol	96-76-4	Acid and chemical	2318 ± 10 (18)	948	950	71.7	0.05 ± 0.0082	0.066 ± 0.0099
ph2	2-Methoxy-4-vinylphenol	7786-61-0	Curry, spice, and vanilla	2188 ± 12 (144)	926	936	63.6	0.034 ± 0.0017	0.058 ± 0.0005
ph3	Phenol	108-95-2	Acid, chemical, dry, ink, medicine, plastic, and rubber	2000 ± 15 (167)	941	941	92.2	n.d.	0.009 ± 0.0004
Phenols	Total							0.084 ± 0.0099	0.133 ± 0.0098
	Others								
ot1	5-Hepten-2-one, 6-methyl-	110-93-0	Banana, citrus, flower, fruit, grass, green, herb, mushroom, and rubber	1338 ± 9 (226)	874	888	95.1	n.d.	0.017 ± 0.0013
ot2	Butanal, 2-methyl-	96-17-3	Almond, cocoa, coffee, fermentation, fruit, and malt	914 ± 8 (126)	829	829	71.2	n.d.	0.073 ± 0.0131
ot3	Dibutyl phthalate	84-74-2	n.f.	2680 ± 13 (12)	952	952	52.2	0.01 ± 0.0006	0.007 ± 0.0004
ot4	Dimethyl phthalate	131-11-3	n.f.	2303 ± 11 (4)	910	910	95.8	0.001 ± 0.0001	n.d.
ot5	Ethyl 9-decenoate	67233-91-4	n.f.	1694 ± 14 (32)	887	935	89	0.034 ± 0.0048	0.049 ± 0.0039
ot6	Ethyl 9-hexadecenoate	54546-22-4	n.f.	2281 ± 12 (14)	927	927	72.2	n.d.	0.041 ± 0.0117
ot7	Isopentyl hexanoate	2198-61-0	Acid, cheese, and sour	1451 ± 5 (29)	886	886	56.6	0.042 ± 0.0013	n.d.
ot8	N-(3-Methylbutyl)acetamide	13434-12-3	n.f.	1866 ± 24 (5)	838	838	93.3	0.01 ± 0.0019	n.d.
ot9	Octane, 1-chloro-	111-85-3	n.f.	1251 ± 9 (7)	919	919	74.2	0.01 ± 0.0019	n.d.
ot10	Dimethyl trisulfide	3658-80-8	Bean, cabbage, garlic, onion, sulfur, and vegetable	1377 ± 11 (163)	932	936	96.8	0.015 ± 0.0117	0.002 ± 0.001
Others	Total							0.121 ± 0.0257	0.188 ± 0.0065

^a^ Volatile flavor compounds detected in the beer. ^b^ Aroma descriptor was derived from the web pages: https://www.flavornet.org/flavornet.html (accessed on 8 May 2026). ^c^ Values are expressed as semi-quantitative concentrations in 4-fluorobenzaldehyde equivalents (μg/L), calculated as (peak area of copound/peak area of internal standard) × concentration of the internal standard. “n.d.”: (not detected) indicates that the compound was not detected in that sample because its target peak was not recognized by mass spectral matching or its experimental retention index deviated excessively from the literature value; n.d. values were treated as zero in the multivariate statistical models. “n.f.”: not found.

**Table A2:** Two-way ANOVA results for structural parameters.

Variable	Factor	*F* Value	*p* Value	Partial *η*^2^
AvgDP (%)	Method	76.76	<0.001	0.906
	Stage	878.6	<0.001	0.991
	Method × Stage	49.75	<0.001	0.861
LowDP (%)	Method	139.68	<0.001	0.946
	Stage	1345.48	<0.001	0.994
	Method × Stage	218.85	<0.001	0.965
HighDP (%)	Method	139.68	<0.001	0.946
	Stage	1345.48	<0.001	0.994
	Method × Stage	218.85	<0.001	0.965
